# Clinical characteristics and renal risk stratification of mixed-type IgA vasculitis: a large-volume study

**DOI:** 10.3389/fimmu.2026.1880224

**Published:** 2026-07-31

**Authors:** Ziyan Li, Shu Huang, Ruiheng Xie, Quxin Juhou, Yunfang Song, Dongqing Wang, Jinxi Zhao, Xiaomin Shi, Wei Zhang, Xiaohong Wang, Jizhen Cai, Xiaowei Tang

**Affiliations:** 1 Department of Gastroenterology, The Affiliated Hospital of Southwest Medical University, Luzhou, China; 2 Department of Gastroenterology, Lianshui County People’ Hospital, Huaian, China; 3 Department of Gastroenterology, Lianshui People’ Hospital of Kangda College Affiliated to Nanjing Medical University, Huaian, China; 4 Department of Gastroenterology, Xuzhou Central Hospital, Xuzhou Clinical School of Xuzhou Medical University, Xuzhou, China; 5 Department of Critical Care Medicine, Xiangya Hospital, Central South University, Changsha, China; 6 Department of Endoscopic Medicine, The Affiliated Hospital of Southwest Medical University, Luzhou, China

**Keywords:** IgA vasculitis, mixed-type IgA vasculitis, proteinuria, renal involvement, retrospective cohort, risk stratification

## Abstract

**Background:**

Mixed-type IgA vasculitis (IgAV) is common in clinical practice, but its internal heterogeneity and prognostic significance, especially in renal involvement, remain unclear. This study aimed to characterize mixed-type IgAV, compare renal risk across mixed subtypes, and evaluate the additional predictive value of mixed subtype classification for clinically significant renal involvement.

**Methods:**

We performed a retrospective cohort study of patients with clinically diagnosed IgAV treated between December 2018 and October 2025. Among 2,818 admissions/records screened, 2,416 unique patients were included after excluding repeated admissions, including 739 non-mixed and 1,677 mixed-type cases. Mixed type and mixed subtypes were defined according to organ involvement at first admission. Clinically significant renal involvement was defined from discharge diagnoses and renal-related clinical documentation, with available renal laboratory indicators used as supporting evidence. Laboratory-based component and sensitivity analyses were additionally performed using predefined proteinuria, eGFR, and serum creatinine criteria. Subtype-specific logistic regression, receiver operating characteristic (ROC) analysis, and a visit-order KM-like proteinuria analysis were performed.

**Results:**

Compared with non-mixed disease, mixed-type IgAV was associated with lower BMI and albumin, higher white blood cell and platelet counts, longer hospital stay, and markedly higher frequencies of digestive, joint, and renal involvement. Within the mixed-type cohort, clinically significant renal involvement varied substantially across subgroups, ranging from 26.7% to 76.9%. Using Skin–Joint as the reference group, significantly higher odds of clinically significant renal involvement were observed in the Other/rare group, Skin–Renal, Skin–Joint–Renal, and Skin–Abdominal–Renal (all *p* < 0.001). In complete-case analysis (n=2,286), adding mixed subtype to a model based on routine demographic and laboratory variables improved the AUC from 0.596 to 0.745 (ΔAUC 0.148, DeLong *p* < 0.001). Mixed-type patients also showed a lower proteinuria-free probability across successive admissions.

**Conclusions:**

Mixed-type IgAV is a heterogeneous phenotype with marked subtype-dependent differences in renal risk. Mixed subtype classification improves identification of clinically significant renal involvement and may support earlier and more individualized renal risk stratification.

## Introduction

IgA vasculitis (IgAV), previously termed Henoch–Schönlein purpura, is a systemic small-vessel vasculitis driven by immune complex deposition in the vessel wall (1). It may occur in both children and adults, although it is more common in the pediatric population (2, 3). In adults, however, the disease is generally less benign and is more likely to be accompanied by renal involvement (4). Clinically, IgAV has traditionally been categorized according to the main organs affected, including cutaneous, abdominal, articular, renal, and mixed forms (5). Mixed-type IgAV, in which more than one system is involved, often presents with diverse and complicated manifestations and therefore remains one of the more difficult aspects of clinical assessment and management (6).

In clinical practice, some patients with IgAV have more than one organ system involved at presentation, most often the gastrointestinal tract, joints, and kidneys (7). This pattern of multisystem involvement can make the diagnosis less straightforward, especially when abdominal or joint symptoms are prominent, and may lead to confusion with other acute abdominal or rheumatic conditions (8). Although current classification criteria for IgAV include purpura together with abdominal, articular, renal, or histopathological features, they do not clearly define what should be considered “mixed-type” disease (9, 10). Consequently, the clinical profile of mixed-type IgAV, the interaction among manifestations across different organs, and their combined influence on prognosis have not yet been fully clarified. As a result, studies specifically addressing this subtype are still limited. The clinical characteristics of mixed-type IgAV, the relationships between different organ manifestations, and their overall impact on prognosis remain insufficiently understood.

Although IgAV has been increasingly studied in recent years, large-volume data from routine clinical practice remain limited, especially for mixed-type disease and renal risk stratification. Epidemiological patterns and infectious triggers of IgAV have also been increasingly discussed (11, 12), but their relationship with organ-combination phenotypes and renal risk remains insufficiently characterized. Therefore, we conducted a retrospective study of patients with clinically diagnosed IgAV treated at a large regional referral center between December 2018 and October 2025. The aims of this study were to describe the clinical features of mixed-type IgAV, summarize the pattern of organ involvement, and identify clinical factors and phenotype combinations associated with renal involvement. Through this, we attempt to provide additional evidence for early recognition of renal-risk phenotypes and more individualized management of mixed-type IgAV.

## Methods

### Study design and setting

This retrospective observational study was conducted at the Affiliated Hospital of Southwest Medical University and included patients with clinically confirmed IgA vasculitis between December 2018 to October 2025. The study was approved by the institutional review board of Affiliated Hospital of Southwest Medical University [Approval no. (KY2025303)]. Due to the retrospective nature of the study, the requirement for informed consent was waived in appropriate circumstances.

### Study population

A total of 2818 hospital admissions/records with clinically confirmed IgAV were identified during the study period. After removal of repeated admissions, 2,416 unique patients were retained for baseline patient-level analyses, including 739 with non-mixed type and 1,677 with mixed type.

Analyses were divided into two levels, but all analyses were derived from the same source period of December 2018 to October 2025. Baseline descriptive analyses, subtype comparisons, and laboratory assessments were performed at the patient level, with the first admission as the baseline record for each patient. By contrast, the visit-order analysis of proteinuria was conducted at the admission-sequence level from the same study period, as repeated admissions were required to assess the accumulation of proteinuria over time. Thus, the distinction between baseline and visit-order analyses was analytical rather than temporal.

### Clinical and laboratory data

The diagnosis of IgAV was based on an established classification framework. In those frameworks, renal involvement is commonly defined as proteinuria greater than 0.3g/24 h and/or abnormal urine red blood cells in the appropriate clinical setting (13, 14).

In the present study, mixed-type IgAV was defined as involvement of at least two organ systems at the first admission. The considered organ domains were the skin, gastrointestinal/abdominal tract, joints, and kidneys. Mixed subtypes were classified according to the baseline combination of organ involvement, including skin–abdominal, skin–joint, skin–renal, skin–abdominal–joint, skin–abdominal–renal, skin–joint–renal, four-system involvement, abdominal–renal, and abdominal–joint. This organ-based classification was used to reflect the multisystem nature of IgAV and to allow comparison of clinically recognizable phenotypes at presentation. A previous adult study of mixed-type Henoch–Schönlein purpura/IgAV also treated mixed disease as a distinct clinical entity defined by multisystem involvement (10).

Clinically significant renal involvement was used in this study as the main outcome for risk stratification and model comparison, because renal disease is widely recognized as the major driver of long-term incidence rate and prognosis in adult IgAV. Recent reviews (15, 16) and the KDIGO 2025 guideline (17) both emphasize that adult IgAV is more severe largely because of renal involvement, and KDIGO discusses the prognosis of IgAV nephritis, which is mainly related to renal function, hypertension, and proteinuria.

Because this was a retrospective cohort, clinically recorded renal involvement was identified primarily from discharge diagnoses and renal-related clinical documentation, with available laboratory data used as supporting evidence and for severity characterization. To improve transparency and comparability, we further evaluated laboratory-based renal abnormalities using a screening definition consisting of any of the following: 24-hour urinary protein ≥0.3 g/24 h, estimated glomerular filtration rate (eGFR) <90 mL/min/1.73 m², or serum creatinine above the sex-specific upper limit of normal. The reason for this choice is that it is consistent with the renal criterion used in established IgAV classification frameworks and the thresholds commonly used in the IgAV literature to represent clinically significant proteinuria (18). However, We recognized that eGFR <90 mL/min/1.73 m² is a sensitive threshold and may overclassify renal abnormality, especially in older adults; therefore, additional sensitivity definitions were constructed by replacing eGFR <90 with eGFR <60 mL/min/1.73 m² and by removing the eGFR criterion entirely. These laboratory definitions were used for component and sensitivity analyses and should not be interpreted as biopsy-proven IgAV nephritis or long-term renal prognosis.

Baseline variables included age, gender, body mass index (BMI), white blood cell count (WBC), platelet count (PLT), albumin, serum creatinine, eGFR, IgA, C-reactive protein (CRP), erythrocyte sedimentation rate (ESR), length of hospital stay, and variables reflecting organ system involvement. Age was summarized both as a continuous variable and using clinically interpretable strata (<7, 7-12, 13-17, 18-59, and ≥60 years).

### Statistical analysis

Within the mixed-type cohort, the proportion of patients with clinically significant renal involvement for each subtype was calculated. Logistic regression was then used to estimate subtype-specific odds ratios (ORs) for renal involvement. The skin–joint subtype was predesignated as the reference category because it represented a mixed phenotype without baseline renal involvement and therefore provided a clinically interpretable comparator for estimating the excess renal risk associated with other mixed subtypes. Using a non-renal mixed subtype as the reference also avoided anchoring comparisons to a group already defined by renal involvement. Very uncommon subtypes were combined into another/rare category to reduce instability of sparse data and improve the robustness of model estimation.

To evaluate whether mixed subtype provided additional value beyond routine baseline variables, two logistic regression models were constructed using baseline complete case data (n=2286). Model 1 included age, sex, albumin, WBC, PLT, BMI, and eGFR. Model 2 included all variables and mixed subtypes in Model 1. Renal involvement was chosen as the binary outcome for ROC analysis because it was the most clinically relevant and consistent prognostic biomarker in this cohort, and because the aim of the ROC comparison was to test whether adding mixed subtype improved identification of patients with a clinically meaningful renal risk characteristics rather than to predict all aspects of long-term outcome (7). Model performance was assessed using receiver operating characteristic (ROC) curves, the area under the curve (AUC), and DeLong testing.

Sensitivity analyses were performed to assess whether the renal-risk pattern was dependent on the relatively sensitive eGFR <90 mL/min/1.73 m² threshold. Two alternative laboratory-based definitions were examined: 24-hour urinary protein ≥0.3 g/24 h, eGFR <60 mL/min/1.73 m², or sex-specific creatinine elevation; and 24-hour urinary protein ≥0.3 g/24 h or sex-specific creatinine elevation without any eGFR criterion. In addition, adjusted logistic models were fitted for each laboratory-based sensitivity definition to estimate the association between mixed-type status and renal abnormality after adjustment for age, sex, albumin, WBC, platelet count, and BMI.

At baseline, albumin, creatinine, eGFR, and CRP were compared between patients with and without renal involvement. Continuous variables were summarized as median and interquartile range (IQR), and categorical variables as counts and percentages. Group comparisons were performed using nonparametric tests for continuous variables and chi-square or Fisher’s exact tests for categorical variables, as appropriate.

In order to explore the dynamic occurrence of proteinuria, further analysis of visit-order was conducted in the 2416-patient cohort. The first proteinuria was defined as the first admission with 24-hour urinary protein ≥0.3 g/24 h. The x-axis of this analysis represents admission sequence rather than standardized calendar follow-up time. Censoring reflects the absence of further hospital records in our institutional database rather than confirmed event-free survival. Therefore, the resulting curve is considered as an exploratory analysis similar to KM, rather than traditional event survival time analysis and should not be used to estimate cumulative incidence in the conventional survival-analysis sense. For patients without renal involvement at baseline who later developed renal involvement during recorded readmissions, the interval from the first admission to the first recorded renal event was summarized descriptively. For graphical presentation only, laboratory values were truncated at the 1st and 99th percentiles. A two-sided p value <0.05 was considered statistically significant. All analyses were performed using R version [4.5.2].

## Results

### Study population and baseline characteristics

**Figure 1:**
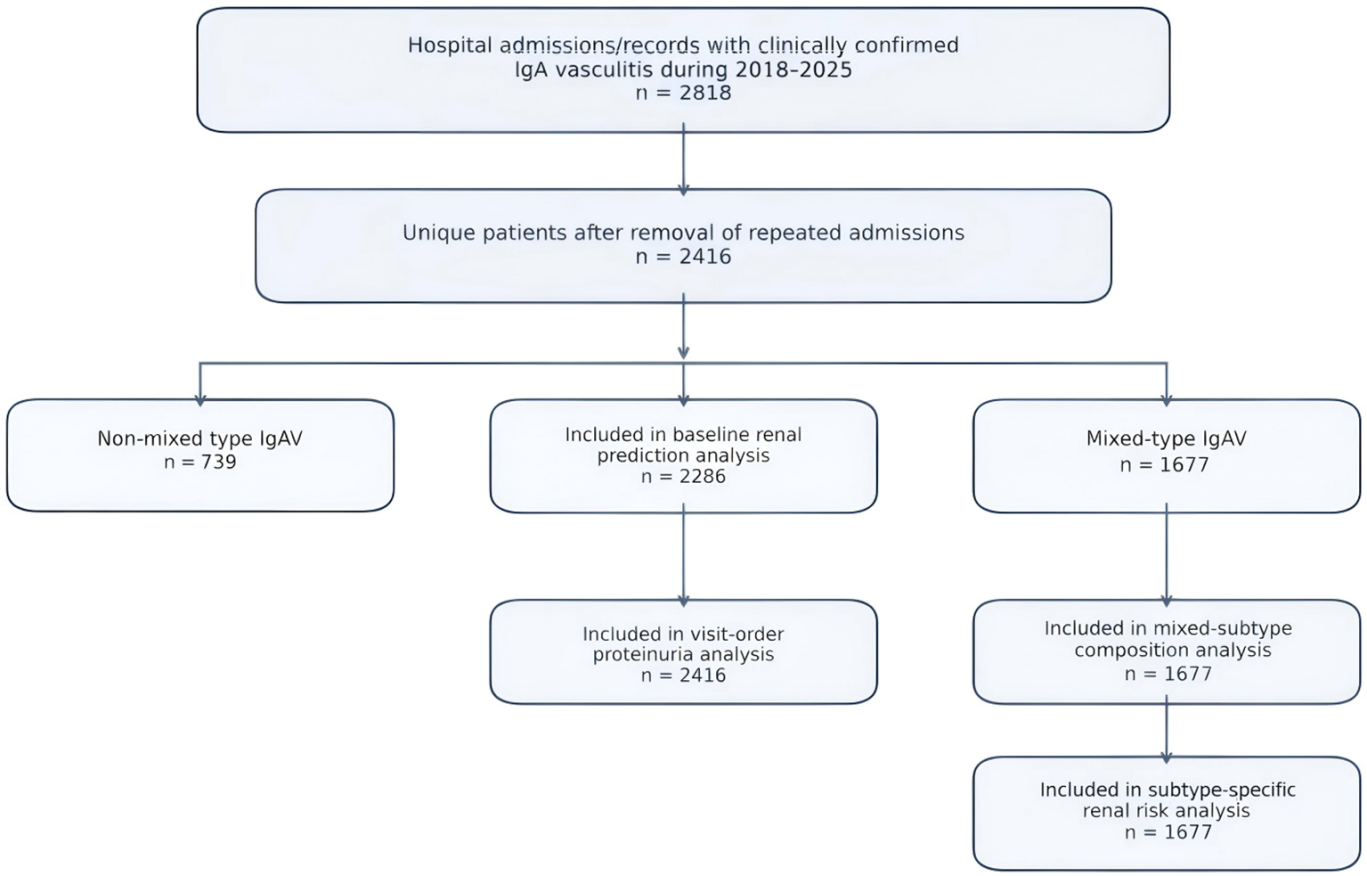
Study flowchart.

As shown in **Figure 1**, a total of 2,818 admissions/records with clinically confirmed IgAV were screened between December 2018 and October 2025. After excluding repeated admissions, 2,416 unique patients were included in the baseline analysis cohort, including 739 non-mixed and 1,677 mixed-type cases.

The study included patients aged 1-89 years. The median age was 10.92 years (IQR, 7.00-18.00). Overall, 1,794 patients (74.3%) were younger than 18 years and 622 patients (25.7%) were adults. More detailed age strata were as follows: <7 years, 585 patients (24.2%); 7-12 years, 811 patients (33.6%); 13-17 years, 398 patients (16.5%); 18-59 years, 439 patients (18.2%); and ≥60 years, 183 patients (7.6%). The cohort included 1,246 males and 1,170 females, corresponding to a male-to-female ratio of 1.06:1.

Baseline characteristics are summarized in **Table 1**.Compared with the non-mixed group, patients with mixed-type IgAV had lower BMI [16.94 (14.87, 21.30) vs 17.78 (15.24, 21.63), p=0.012], higher WBC [10.27 (7.96, 13.76) vs 8.86 (6.62, 11.96), p<0.001], higher platelet count [325.00 (261.00, 402.00) vs 302.00 (239.00, 368.50), p<0.001], lower albumin [41.80 (38.00, 44.60) vs 42.60 (39.70, 45.30), p<0.001], and a slightly longer length of hospital stay [7.00 (5.00, 9.00) vs 6.00 (5.00, 8.00) days, p=0.025]. No significant differences were observed for age, creatinine, eGFR, IgA, CRP, or ESR.

**Table 1:** Baseline characteristics of patients with non-mixed and mixed-type IgA vasculitis.

Variable	Non-mixed (n=739)	Mixed (n=1677)	P value
Age (years)	10.67 (7.08, 21.00)	11.17 (7.00, 17.00)	0.821
BMI (kg/m²)	17.78 (15.24, 21.63)	16.94 (14.87, 21.30)	0.012
WBC	8.86 (6.62, 11.96)	10.27 (7.96, 13.76)	<0.001
PLT	302.00 (239.00, 368.50)	325.00 (261.00, 402.00)	<0.001
Albumin (g/L)	42.60 (39.70, 45.30)	41.80 (38.00, 44.60)	<0.001
Creatinine (µmol/L)	42.50 (32.25, 57.20)	42.40 (31.80, 58.40)	0.637
eGFR (mL/min/1.73m²)	157.80 (126.70, 180.90)	159.95 (131.20, 184.33)	0.280
IgA	2.63 (1.99, 3.52)	2.70 (2.04, 3.56)	0.847
CRP	5.70 (2.10, 19.70)	6.35 (2.38, 20.78)	0.816
ESR	18.00 (7.75, 32.00)	20.00 (10.00, 36.75)	0.462
Hospital stay (days)	6.00 (5.00, 8.00)	7.00 (5.00, 9.00)	0.025
Male	349/739 (47.2%)	897/1677 (53.5%)	0.005
Skin involvement	612/739 (82.8%)	1657/1677 (98.8%)	<0.001
Digestive involvement	95/739 (12.9%)	1147/1677 (68.4%)	<0.001
Joint involvement	3/739 (0.4%)	625/1677 (37.3%)	<0.001
Renal involvement	16/739 (2.2%)	595/1677 (35.5%)	<0.001
System count ≥3	0/739 (0.0%)	593/1677 (35.4%)	<0.001

In terms of categorical variables, mixed-type patients were more often male than non-mixed patients (53.5% vs 47.2%, p=0.005) and had substantially higher frequencies of skin involvement (98.8% vs 82.8%), digestive involvement (68.4% vs 12.9%), joint involvement (37.3% vs 0.4%), clinically significant renal involvement (35.5% vs 2.2%), and involvement of at least three organ systems (35.4% vs 0.0%) (all p<0.001).

### Mixed-type subgroups, renal risk, and predictive performance

**Figure 2:**
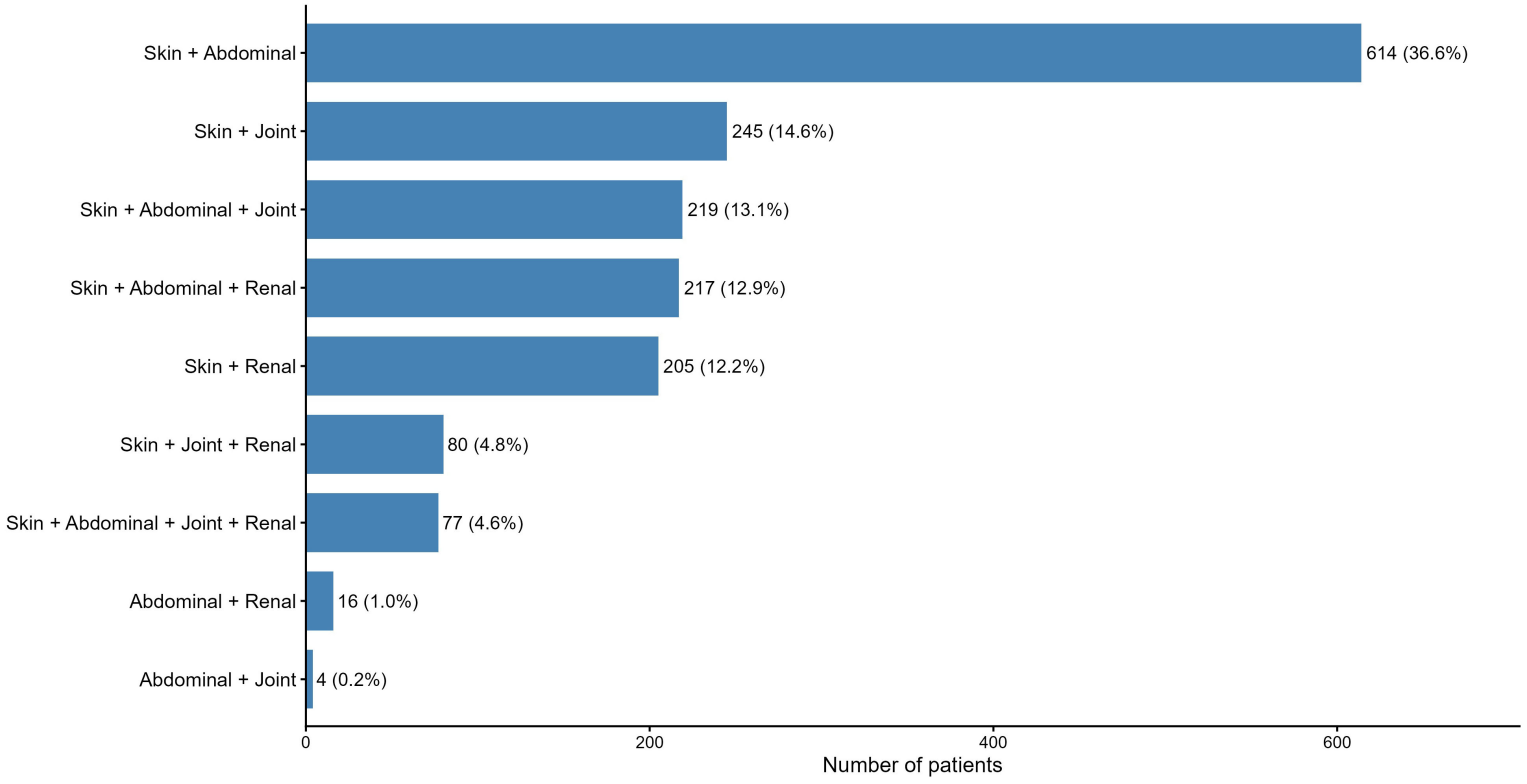
Composition of organ involvement patterns in mixed-type IgAV. IgAV, IgA vasculitis.

Among the 1,677 patients with mixed-type IgAV, the most common pattern of organ involvement was Skin–Abdominal (n=614), followed by Skin–Joint (n=245), Skin–Abdominal–Joint (n=219), Skin–Abdominal–Renal (n=217), Skin–Renal (n=205), Skin–Joint–Renal (n=80), and four-system involvement (n=77). Abdominal–Renal (n=16) and Abdominal–Joint (n=4) were uncommon (**Figure 2**).

**Figure 3:**
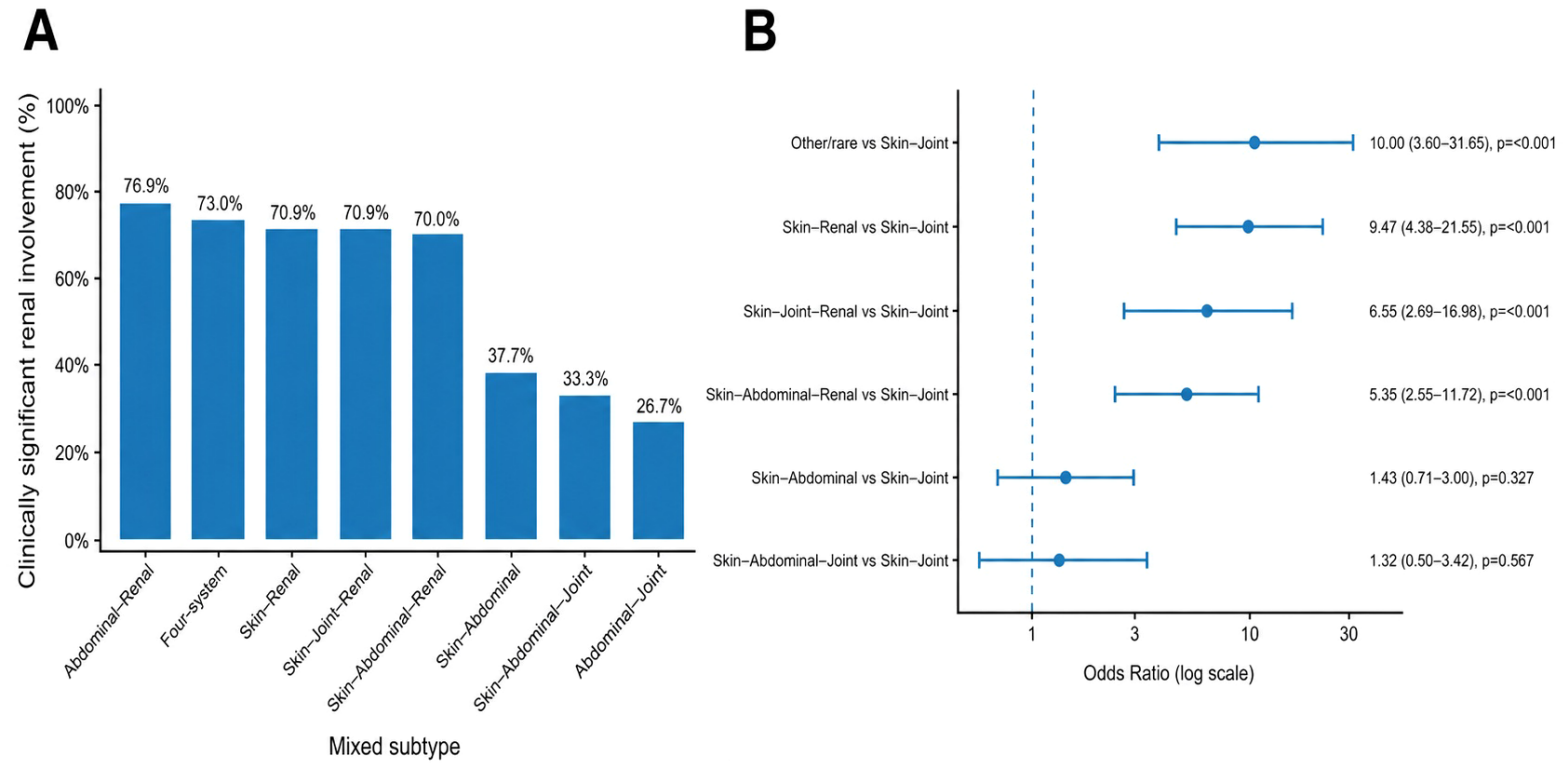
Renal involvement across mixed-type subgroups. **(A)** Proportion of clinically significant renal involvement across mixed-type subgroups. **(B)** Subtype-specific odds ratios for clinically significant renal involvement using Skin–Joint as the reference subgroup.

In the mixed-type subgroups, there was a significant difference in the frequency of clinically significant renal involvement (**Figure 3A**). The highest proportions were observed in Abdominal–Renal (76.9%), four-system involvement (73.0%), Skin–Renal (70.9%), Skin–Joint–Renal (70.9%), and Skin–Abdominal–Renal (70.0%). In contrast, lower proportions were seen in Skin–Abdominal (37.7%), Skin–Abdominal–Joint (33.3%), and Skin–Joint (26.7%). Because Abdominal–Joint cases were extremely rare, this subgroup was not considered suitable for stable interpretation.

Using Skin–Joint as the reference group, logistic regression further showed significant differences in renal risk across different subtypes (**Figure 3B**). The odds of clinically significant renal involvement were significantly higher in the Other/rare group (OR 10.00, 95% CI 3.60–31.65, p<0.001), Skin–Renal (OR 9.47, 95% CI 4.38–21.55, p<0.001), Skin–Joint–Renal (OR 6.55, 95% CI 2.69–16.98, p<0.001), and Skin–Abdominal–Renal (OR 5.35, 95% CI 2.55–11.72, p<0.001). By comparison, Skin–Abdominal (OR 1.43, 95% CI 0.71–3.00, p=0.327) and Skin–Abdominal–Joint (OR 1.32, 95% CI 0.50–3.42, p=0.567) did not differ significantly from Skin–Joint.

Univariable logistic regression analyses are presented in **Table 2**. In crude analyses, mixed-type status, lower albumin, lower eGFR, and several baseline laboratory variables were significantly associated with clinically significant renal involvement. For prolonged hospital stay (≥7 days), the overall pattern is different, and inflammation and renal function related markers show stronger associations than individual phenotype classifications.

**Table 2:** Univariable logistic regression analyses for clinically significant renal involvement and prolonged hospital stay (≥7 days).

Outcome	Predictor	N	OR	95% CI	P
Renal involvement (in-hospital)	Age (years)	2416	1.01	1.01–1.01	<0.001
Renal involvement (in-hospital)	Male	2416	1.04	0.87–1.25	0.647
Renal involvement (in-hospital)	Albumin (g/L)	2392	0.93	0.91–0.95	<0.001
Renal involvement (in-hospital)	Creatinine (µmol/L)	2394	1.00	1.00–1.01	<0.001
Renal involvement (in-hospital)	eGFR	2395	0.99	0.99–1.00	<0.001
Renal involvement (in-hospital)	IgA	450	0.95	0.82–1.10	0.520
Renal involvement (in-hospital)	CRP	341	1.00	0.99–1.00	0.536
Renal involvement (in-hospital)	ESR	202	1.01	0.99–1.02	0.239
Renal involvement (in-hospital)	Mixed type	2416	24.85	15.52–42.92	<0.001
Renal involvement (in-hospital)	BMI (kg/m²)	2319	1.00	1.00–1.01	0.525
Renal involvement (in-hospital)	WBC	2405	1.01	0.99–1.03	0.188
Renal involvement (in-hospital)	PLT	2405	1.00	1.00–1.00	0.023
Prolonged stay (≥7 days)	Age (years)	802	1.06	1.01–1.02	<0.001
Prolonged stay (≥7 days)	Male	802	1.47	1.11–1.95	0.007
Prolonged stay (≥7 days)	Albumin (g/L)	791	0.91	0.88–0.93	<0.001
Prolonged stay (≥7 days)	Creatinine (µmol/L)	792	1.01	1.01–1.02	<0.001
Prolonged stay (≥7 days)	eGFR	792	0.99	0.99–1.00	<0.001
Prolonged stay (≥7 days)	IgA	176	1.00	0.80–1.24	0.988
Prolonged stay (≥7 days)	CRP	139	1.03	1.01–1.05	0.003
Prolonged stay (≥7 days)	ESR	93	1.01	0.99–1.03	0.282
Prolonged stay (≥7 days)	Mixed type	802	1.35	1.01–1.81	0.040
Prolonged stay (≥7 days)	BMI (kg/m²)	766	1.00	1.00–1.01	0.452
Prolonged stay (≥7 days)	WBC	797	1.06	1.03–1.09	<0.001
Prolonged stay (≥7 days)	PLT	797	1.00	1.00–1.00	0.629

Multivariable analyses are shown in **Table 3**. After adjusting for age, sex, albumin, WBC, PLT, BMI, and eGFR, mixed-type status remained strongly associated with clinically significant renal involvement. Lower albumin also remained an independent factor associated with renal involvement. In contrast, in the adjusted model for prolonged hospital stay, mixed-type status was not independently significant, whereas males, higher WBC, and lower eGFR remained associated with longer hospitalization.

**Table 3:** Multivariable logistic regression analyses for clinically significant renal involvement and prolonged hospital stay (≥7 days).

Outcome	Predictor	OR	95% CI	P
Renal involvement (in-hospital)	Mixed type (vs non-mixed)	23.88	14.84–41.40	<0.001
Renal involvement (in-hospital)	Age (per 1 year)	1.00	0.99–1.00	0.350
Renal involvement (in-hospital)	Male (vs female)	1.07	0.86–1.33	0.559
Renal involvement (in-hospital)	Albumin (per 1 g/L)	0.95	0.93–0.97	<0.001
Renal involvement (in-hospital)	eGFR (per 1 unit)	1.00	0.99–1.00	0.037
Renal involvement (in-hospital)	WBC (per 1 unit)	1.00	0.98–1.01	0.625
Renal involvement (in-hospital)	BMI (per 1 kg/m²)	1.00	0.99–1.01	0.633
Renal involvement (in-hospital)	PLT (per 1 unit)	1.00	1.00–1.00	0.008
Prolonged stay (≥7 days)	Mixed type (vs non-mixed)	1.24	0.91–1.69	0.181
Prolonged stay (≥7 days)	Age (per 1 year)	1.00	0.99–1.02	0.500
Prolonged stay (≥7 days)	Male (vs female)	1.46	1.07–2.00	0.019
Prolonged stay (≥7 days)	Albumin (per 1 g/L)	0.94	0.90–0.97	<0.001
Prolonged stay (≥7 days)	eGFR (per 1 unit)	1.00	0.99–1.00	0.392
Prolonged stay (≥7 days)	WBC (per 1 unit)	1.05	1.01–1.08	0.014
Prolonged stay (≥7 days)	BMI (per 1 kg/m²)	1.00	1.00–1.01	0.772
Prolonged stay (≥7 days)	PLT (per 1 unit)	1.00	1.00–1.00	0.779

**Figure 4:**
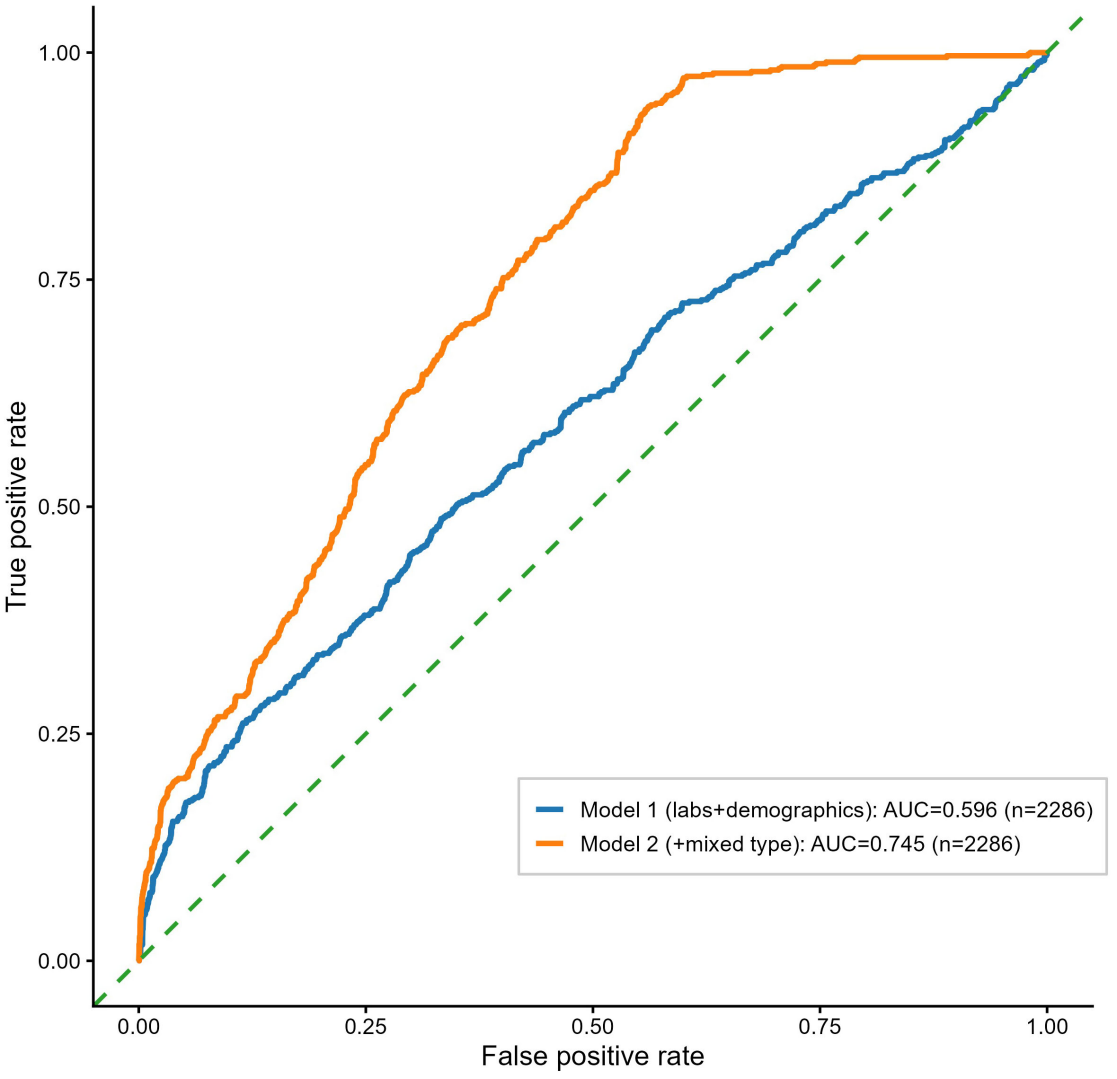
ROC curves for predicting renal involvement. Model 1 included age, sex, albumin, white blood cell count, platelet count, BMI, and eGFR. Model 2 additionally incorporated mixed subtype; ROC, receiver operating characteristic.

In the baseline complete case analysis (n=2286), the AUC of Model 1 (including age, sex, albumin, WBC, PLT, BMI, and eGFR) was 0.596 (95% confidence interval 0.568-0.624). After adding the mixed subtype, Model 2 achieved an AUC of 0.745 (95% CI 0.724–0.766), corresponding to a ΔAUC of 0.148, with a significant improvement by DeLong testing (p<0.001) (**Figure 4**; **Supplementary Table 1**). These findings suggest that mixed subtype provided meaningful additional information for identifying clinically significant renal involvement affected patients beyond routine baseline demographic and laboratory variables.

### Renal involvement–related laboratory differences and visit-order proteinuria pattern

**Figure 5:**
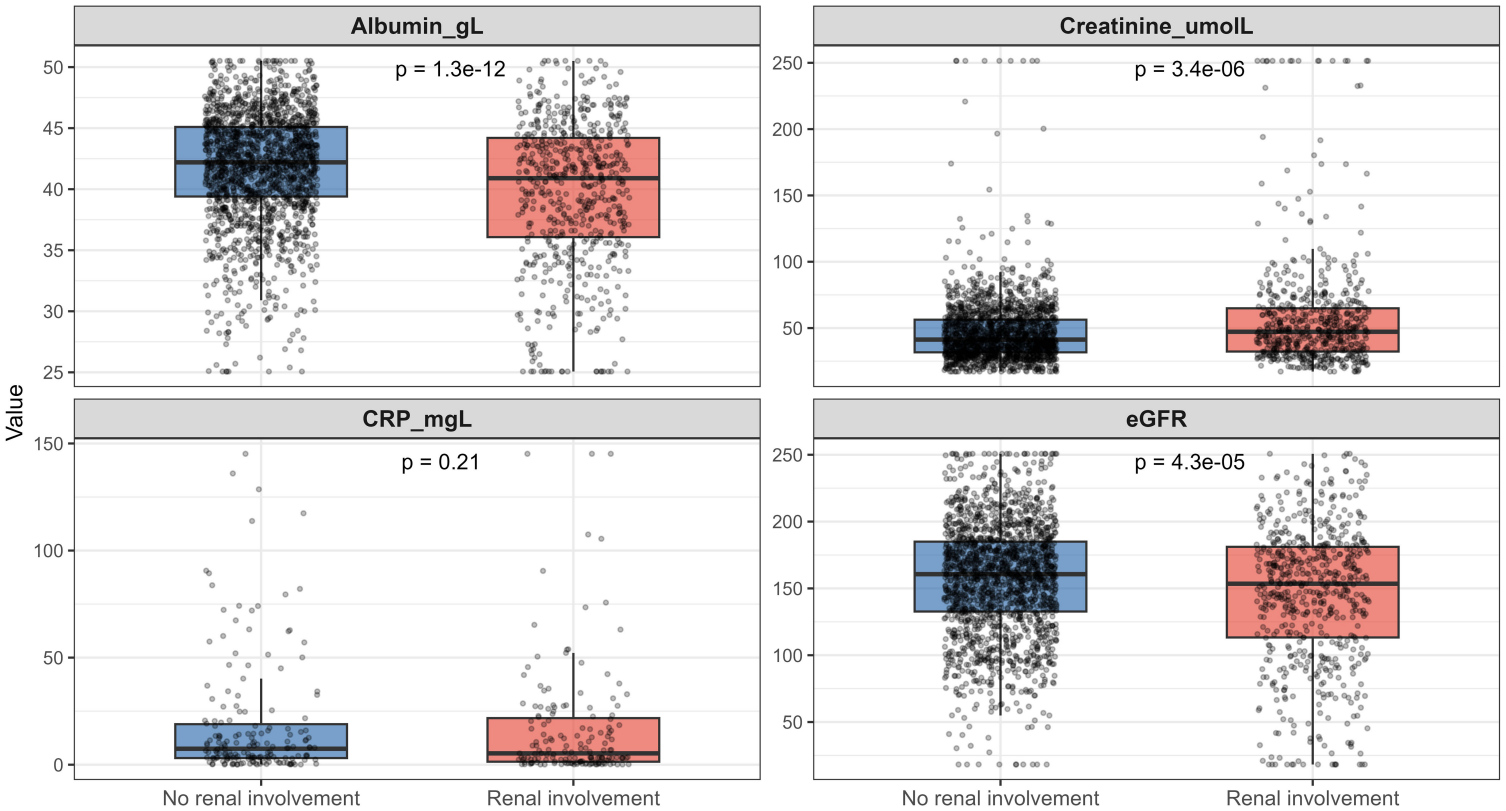
Comparison of key laboratory markers by renal involvement.

At baseline, patients with renal involvement had lower albumin [40.90 (36.08, 44.20) vs 42.20 (39.40, 45.10) g/L, P<0.001], higher creatinine [47.20 (32.30, 64.90) vs 41.30 (31.70, 56.30) μmol/L, P<0.001], and lower eGFR [153.50 (113.30, 181.10) vs 160.65 (132.70, 184.97), P<0.001] than those without renal involvement. CRP levels were comparable between the two groups [5.30 (1.40, 21.75) vs 7.45 (3.11, 18.92) mg/L, P = 0.203] (**Figure 5**; **Supplementary Table 2**). In the baseline cohort, eGFR data were available for 2,395 patients. Overall, 200 patients (8.3%) had eGFR <90 mL/min/1.73 m², while more severe renal dysfunction was uncommon, with eGFR <60, <30, and <15 mL/min/1.73 m² observed in 76 (3.1%), 32 (1.3%), and 17 (0.7%) patients, respectively. Although patients with renal involvement showed lower eGFR levels overall, the median values in both groups remained relatively preserved, indicating that most renal abnormalities were identified at an early stage.

The component and sensitivity analyses of laboratory-defined renal involvement are summarized in **Supplementary Table 3**. In the baseline cohort of 2,416 patients, 328 (13.6%) met the proteinuria criterion, 200 (8.3%) met the eGFR <90 criterion, and 78 (3.2%) the sex-specific creatinine criterion. In total, 431 patients (17.8%) fulfilled at least one of these criteria. Among them, 76 patients (3.1%) were classified solely based on eGFR <90 without accompanying proteinuria or elevated creatinine. Under stricter definitions using eGFR <60 or removing the eGFR criterion entirely, 361 (14.9%) and 355 (14.7%) patients met the renal abnormality definition, respectively. In adjusted sensitivity models, mixed-type status remained significantly associated with renal abnormality under all three definitions, indicating that the observed renal-risk pattern was not driven solely by the eGFR <90 threshold.

**Figure 6:**
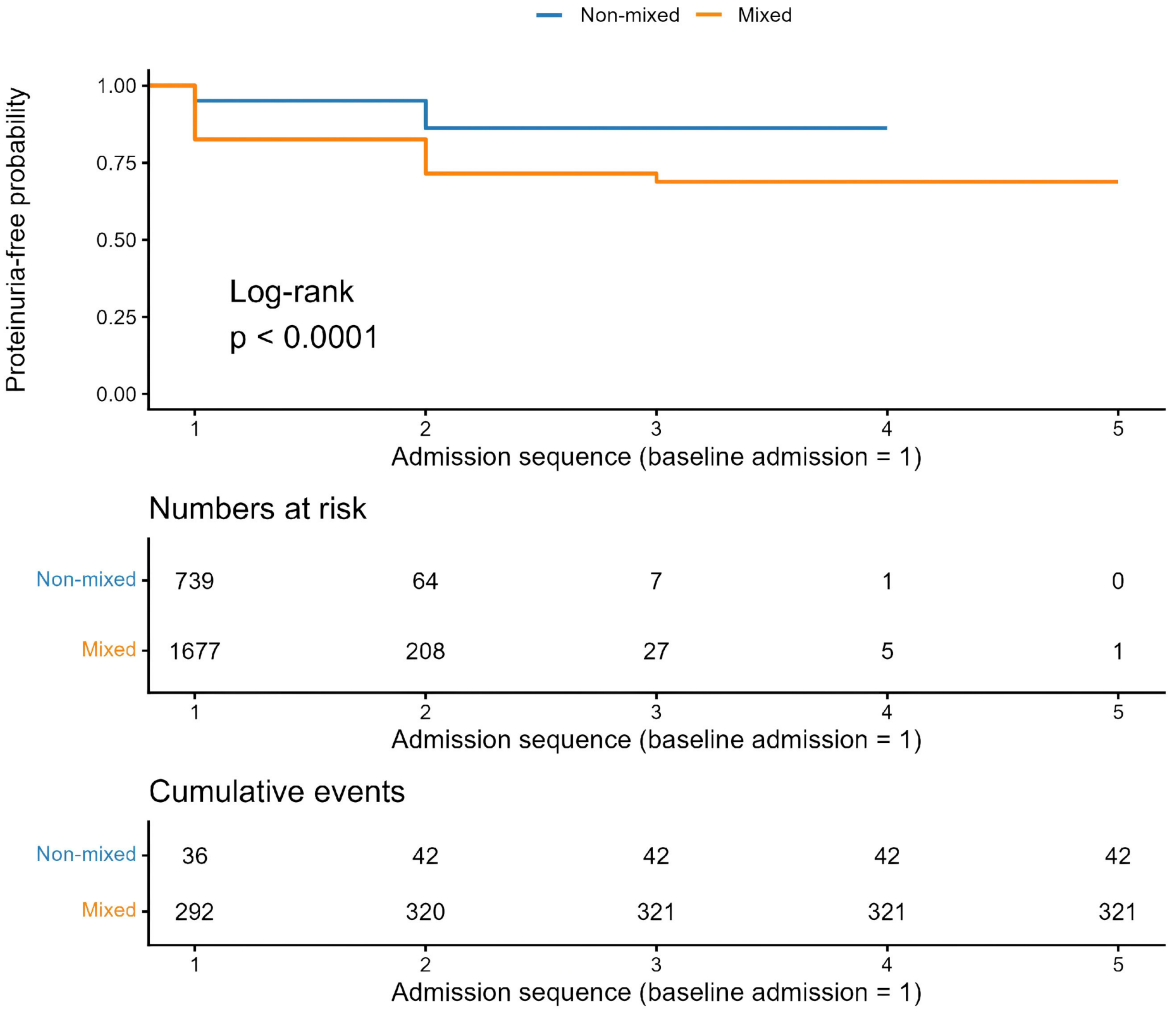
KM-like curves for first proteinuria across admission sequence.

In the visit-order analysis, mixed-type patients showed a consistently lower proteinuria-free probability across successive admissions than non-mixed patients (**Figure 6**). The curves separated in the early and remained apart across recorded admissions, with a significant log-rank test (p<0.0001). The numbers at risk decreased markedly after the first admission, from 739 to 64 in the non-mixed group and from 1,677 to 208 in the mixed-type group, indicating that later admissions reflected a selected subset of readmitted patients. Among the 334 patients who experienced at least one readmission, the median interval between the first and second admissions was 22.5 days (IQR, 10.0–75.8). For patients without renal involvement at baseline who later developed renal involvement, the median time to the first recorded renal event was 32.0 days (IQR, 17.0–84.5). Since this analysis was based on admission sequence rather than standardized follow-up time, it should be interpreted as an exploratory rather than as conventional survival or cumulative incidence analysis.

Additional analyses are presented in the **Supplementary Material**. In short, mixed-type IgAV was associated with relatively high rates of abnormal inflammatory and immune indices, particularly CRP and ESR, whereas abnormal IgA levels and hypoalbuminemia were less frequent (**Supplementary Figure 1**). Overall, the supplementary multivariable analyses were consistent with the main findings and did not materially alter the interpretation of the results (**Supplementary Figures 2**, **3**).

## Discussion

In this retrospective cohort study, mixed-type IgAV was more common than non-mixed disease and showed marked clinical heterogeneity. Compared with non-mixed IgAV, mixed-type disease was associated with broader organ involvement and a substantially higher frequency of clinically significant renal involvement. More importantly, mixed-type IgAV was not a uniform category. Within this group, baseline organ involvement patterns were associated with clearly different renal risk characteristics, with renal-containing phenotypes showing the highest frequencies and odds of renal involvement. In addition, adding mixed subtype to a baseline model that included routine demographic and laboratory variables significantly improved discrimination for clinically significant renal involvement. In summary, these findings suggest that mixed-type IgAV may be better understood as a clinically stratifiable phenotype rather than a single descriptive subgroup.

This interpretation is consistent with the current understanding of IgAV pathogenesis (19, 20). IgAV is thought to be caused by abnormal IgA-mediated immune responses, including the formation of circulating immune complexes, followed by complement activation, leukocyte recruitment, endothelial injury, and increased vascular permeability (21, 22). These processes can affect multiple vascular beds, including the skin, gastrointestinal tract, joints, and kidneys. Therefore, mixed-type IgAV may reflect a broader systemic small-vessel vasculitis phenotype rather than a simple coexistence of several symptoms. Patients with three- or four-system involvement may have a greater systemic inflammatory and vascular burden, this may help explain why renal abnormalities were more frequent in mixed-type disease. Even when renal involvement was not clinically apparent at the first admission, extra-renal organ involvement may indicate an active systemic vasculitic process with the potential to affect the kidney later. We have therefore interpreted these findings as clinically meaningful associations rather than direct evidence of causality.

From a clinical and nephrology standpoint, the value of mixed-type classification lies in identifying patients who may require closer renal surveillance. This is important as renal involvement remains the most significant determinant of long-term IgAV outcomes (23), especially in adults (24). Our data suggest that two patients may both meet the definition of mixed-type disease while having very different renal risk, depending on which organs are involved at baseline. Patients with renal-containing mixed subtypes, reduced eGFR, elevated creatinine, proteinuria, hypoalbuminemia, hypertension, or three-/four-system involvement should therefore be considered high risk and should receive early nephrology evaluation and close renal monitoring, which consistent with Current guidelines (25). Recent studies (26) also emphasize the importance of proteinuria levels, renal function, and pathological findings in predicting prognosis. Accordingly, mixed subtype classification should not replace standard renal evaluation or biopsy when indicated, but may serve as an early indicator to guide closer monitoring and referral.

The ROC and sensitivity analysis points in the same direction. After adding mixed subtypes to a model that includes age, sex, albumin, WBC, PLT, BMI, and eGFR, the ability to distinguish clinically significant renal involvement is greatly improved. This suggests that clinical phenotype captures information that is not fully reflected by conventional laboratory markers. That possibility is reasonable in view of the current literature (27), where several inflammatory, immune, and endothelial markers have been explored in IgAV, but none has become sufficiently reliable to replace careful clinical assessment for renal risk stratification. While lower albumin, higher creatinine, and lower eGFR are expected in patients with renal involvement, the main contribution of this study is that mixed-type classification adds further predictive value and improved renal risk stratification. Moreover, Sensitivity analyses also showed that the observed associations were not solely driven by the relatively sensitive eGFR threshold of <90 mL/min/1.73 m².

The laboratory findings were also consistent with the renal-risk pattern observed in the main analyses. Patients with clinically significant renal involvement had lower albumin, higher creatinine, and lower eGFR at baseline, whereas CRP showed no significant difference between renal and non-renal groups. Lower albumin remained independently associated with renal involvement, including in the supplementary analysis limited to mixed-type patients. This association is not difficult to understand clinically. In IgAV, low albumin may reflect urinary protein loss, increased vascular permeability, and overall disease burden rather than nutritional status alone (28). By contrast, inflammatory markers such as CRP and ESR were frequently abnormal in mixed-type disease overall but appeared less specific for renal involvement itself. That may explain why inflammatory-marker abnormalities were common in mixed-type disease overall, while albumin showed a closer relationship with renal involvement in the main models.

The relationship between organ combinations and renal risk may also have a pathophysiologic basis. IgAV is fundamentally a systemic small-vessel vasculitis, and immune-complex deposition with downstream complement activation has been described not only in the kidney but also in skin lesions (29, 30). Complement pathway activation in skin lesions supports the idea that skin, gastrointestinal, and renal manifestations may represent different expressions of a shared vascular inflammatory process rather than unrelated complications (31). Therefore, in our study, it appears more reasonable from this perspective that certain mixed phenotypes have a higher renal risk.

The visit-order proteinuria analysis offers a different but exploratory angle on the same problem. Mixed-type patients showed a lower proteinuria-free probability across successive admissions, with earlier curve separation. Among those who initially had no renal involvement but later developed it, the first renal event occurred at a median of about one month. This finding highlights the importance of early monitoring after diagnosis. Although this pattern was directionally consistent with the baseline renal findings, it does not represent conventional survival analysis as the x-axis represents admission sequence rather than standardized follow-up time. Additionally, the number of patients decreased substantially after the second admission, limiting the robustness of later estimates. Therefore, this analysis supports the need for early renal surveillance but should not be interpreted as a conventional cumulative-incidence estimate. Even so, the early separation of the curves is consistent with the possibility that mixed-type disease, particularly renal-containing phenotypes, reflects a heavier burden of systemic vascular injury from the beginning (32).

Another point of interest is that the factors associated with renal involvement were not identical to those associated with prolonged hospitalization. Mixed-type status remained independently associated with clinically significant renal involvement, but not with longer hospital stay after adjustment. On the contrary, male, higher WBC count, and lower eGFR were more closely related to prolonged hospitalization. This suggests that renal risk and short-term inpatient burden may represent overlapping but distinct dimensions of disease severity. The observed sex effect is also noteworthy, as new evidence suggests that renal involvement in IgAV may be more severe in males (33).

Based on these findings and current monitoring recommendations17,18, a practical follow-up strategy can be proposed. All patients with mixed-type IgAV should undergo baseline evaluation, including urinalysis, urine protein measurement, urine microscopy, blood pressure, serum creatinine, eGFR, and albumin. For those without initial renal involvement, repeat urine testing and blood pressure monitoring may be performed weekly during the first month, every two weeks from weeks 5 to 12, monthly until 6 months, and again at one year. Patients with renal-involved subtypes, multiple system involvement, male sex, low albumin, recurrent symptoms, gastrointestinal involvement, hypertension, proteinuria, hematuria, reduced eGFR, or elevated creatinine may need closer observation and earlier nephrology consultation. Decisions regarding biopsy or treatment should be guided by renal findings and established guidelines rather than mixed subtype alone.

## Strengths and limitations

The main strength of this study is its large hospital-based cohort, which allowed us to characterize mixed-type IgAV and evaluate the additional value of organ-combination phenotypes for renal risk stratification in routine clinical practice. However, Several limitations need to be kept in mind. As a retrospective analysis from a single center, the findings mainly reflect institutional referral experience rather than population incidence. Renal involvement was determined from available clinical records and routine laboratory indicators, rather than uniformly confirmed by renal biopsy. In addition, detailed information on biopsy pathology, treatment regimens, and dialysis was not consistently recorded in a structured variables, which limits further evaluation of pathological features and treatment effects. The proteinuria analysis based on admission sequence should be interpreted with caution, as it relied on repeated hospitalization records instead of standardized follow-up time. Moreover, some mixed subgroups included relatively few patients, which may affect the robustness of subgroup comparisons. Future multicenter prospective studies with more comprehensive clinical and pathological data are needed to further validate these findings.

## Conclusion

In conclusion, mixed-type IgAV should not be regarded as a single uniform clinical category. Instead, it represents a heterogeneous phenotype in which baseline patterns of organ involvement are associated with significantly different renal risks. Compared with non-mixed disease, mixed-type IgAV has broader multisystem involvement and a substantially higher frequency of clinically significant renal involvement. Within the mixed-type cohort, renal-containing subtypes carried the greatest renal burden, and inclusion of mixed subtype improved risk discrimination beyond conventional demographic and laboratory variables. Component and sensitivity analyses supported the robustness of this renal-risk pattern, while also emphasizing that laboratory thresholds such as eGFR <90 mL/min/1.73 m² should be interpreted cautiously. These findings support a phenotype-based approach to early renal risk assessment in IgAV. Mixed-type patients, including those without apparent renal involvement at baseline, should receive structured renal monitoring after diagnosis, and high-risk phenotypes should be considered for closer follow-up and early nephrology evaluation. Further prospective multicenter studies with standardized pathology and treatment data are needed to validate this proposed stratification and follow-up strategy.

## Data Availability

The dataset contains clinical information from human participants and is restricted due to patient privacy and ethical requirements. The data are not publicly available. De-identified data may be made available from the corresponding author upon reasonable request, subject to institutional approval and applicable ethical regulations. Requests to access these datasets should be directed to Ziyan Li, 1277973002@qq.com.

## Supplementary material

Supplementary Figure 1 Abnormal Rates of Inflammatory and Immune Markers in Mixed-type IgAV. IgAV, IgA vasculitis.

Supplementary Figure 2 Multivariable Logistic Regression for Renal Involvement (Mixed-type Only).

Supplementary Figure 3 Multivariable Logistic Regression for Prolonged Stay(≥7days).

Supplementary Table 1 Discrimination performance of prediction models for renal involvement.

Supplementary Table 2 Baseline comparison of key laboratory markers according to clinically significant renal involvement.

Supplementary Table 3 Component counts, sensitivity definitions, and adjusted mixed-type associations for laboratory-based renal abnormality definitions.
