# Supplementary material for: Clinical characteristics and renal risk stratification of mixed-type IgA vasculitis: a large-volume study
Source: Front Immunol. 2026 Jul 31;17:1880224. doi: 10.3389/fimmu.2026.1880224 (PMC13473345; doi:10.3389/fimmu.2026.1880224)
Supplement: Supplementary file 1 [file SupplementaryFile1.docx]

| **Supplementary Table 1** Discrimination performance of prediction models for renal involvement. | | | | |
| --- | --- | --- | --- | --- |
| **Model** | **N** | **AUC_CI** | **Delta_AUC** | **DeLong_p** |
| Model 1: Labs + demographics | 2,286 | 0.596 (0.568–0.624) |  |  |
| Model 2: Model 1 + mixed subtype | 2,286 | 0.745 (0.724–0.766) | 0.148 | <0.001 |
