# Supplementary material for: Clinical characteristics and renal risk stratification of mixed-type IgA vasculitis: a large-volume study
Source: Front Immunol. 2026 Jul 31;17:1880224. doi: 10.3389/fimmu.2026.1880224 (PMC13473345; doi:10.3389/fimmu.2026.1880224)
Supplement: Supplementary file 2 [file SupplementaryFile2.docx]

| **Supplementary Table 2** Baseline comparison of key laboratory markers according to clinically significant renal involvement. | | | |
| --- | --- | --- | --- |
| Marker | No renal involvement | Renal involvement | P |
| Albumin, g/L | 42.20 (39.40, 45.10) [n=1784] | 40.90 (36.08, 44.20) [n=608] | <0.001 |
| Creatinine, umol/L | 41.30 (31.70, 56.30) [n=1785] | 47.20 (32.30, 64.90) [n=609] | <0.001 |
| eGFR | 160.65 (132.70, 184.97) [n=1786] | 153.50 (113.30, 181.10) [n=609] | <0.001 |
| CRP, mg/L | 7.45 (3.11, 18.92) [n=178] | 5.30 (1.40, 21.75) [n=163] | 0.203 |
