# Supplementary material for: Clinical characteristics and renal risk stratification of mixed-type IgA vasculitis: a large-volume study
Source: Front Immunol. 2026 Jul 31;17:1880224. doi: 10.3389/fimmu.2026.1880224 (PMC13473345; doi:10.3389/fimmu.2026.1880224)
Supplement: Supplementary file 3 [file SupplementaryFile3.docx]

| **Supplementary Table 3** Components and sensitivity definitions of renal involvement | | | |
| --- | --- | --- | --- |
| **Category** | **Definition / criterion** | **N** | **%** |
| Primary laboratory renal abnormality definition | 24-hour urinary protein ≥0.3 g/24 h, or eGFR <90 mL/min/1.73 m², or serum creatinine above the sex-specific upper limit of normal | 431 / 2416 | 17.8 |
| Individual criterion | 24-hour urinary protein ≥0.3 g/24 h | 328 / 2416 | 13.6 |
| Individual criterion | eGFR <90 mL/min/1.73 m² | 200 / 2416 | 8.3 |
| Individual criterion | Serum creatinine above the sex-specific upper limit of normal | 78 / 2416 | 3.2 |
| eGFR-related classification | eGFR <90 mL/min/1.73 m² without 24-hour urinary protein ≥0.3 g/24 h | 101 / 2416 | 4.2 |
| eGFR-related classification | Isolated eGFR <90 mL/min/1.73 m² alone, without proteinuria or elevated creatinine | 76 / 2416 | 3.1 |
| Sensitivity definition 1: stricter eGFR threshold | 24-hour urinary protein ≥0.3 g/24 h, or eGFR <60 mL/min/1.73 m², or serum creatinine above the sex-specific upper limit of normal | 361 / 2416 | 14.9 |
| Sensitivity definition 2: without eGFR criterion | 24-hour urinary protein ≥0.3 g/24 h, or serum creatinine above the sex-specific upper limit of normal | 355 / 2416 | 14.7 |

Baseline patient-level cohort, first admission only (n = 2,416).
